# Correction: Ribovirus classification by a polymerase barcode sequence

**DOI:** 10.7717/peerj.14055/correction-1

**Published:** 2024-08-09

**Authors:** Artem Babaian, Robert Edgar

**Affiliations:** 1 St Edmunds College, Cambridge, United Kingdom; 2 Department of Haematology, University of Cambridge, Cambridge, United Kingdom; 3 Corte Madera, California, United States of America

Corrigendum:

After consultation with the Section Editors for the “Bioinformatics and Genomics” Section and in agreement with the Authors, Figure 3 (and its associated caption) has been corrected.

Original PDF

