# Supplementary material for: Ribovirus classification by a polymerase barcode sequence
Source: PeerJ. 2022 Oct 13;10:e14055. doi: 10.7717/peerj.14055 (PMC9573346; doi:10.7717/peerj.14055)
Supplement: Supplemental Information 1 — PalmID search results include hits to SRA accessions, word cloud with keywords, visualization of palmscan alignment and links to downloadable tables. [file peerj-10-14055-s001.zip › ruby.html]

palmID: RNA Virus RdRP Report


Code 

- Show All Code
- Hide All Code
- Download Rmd

# palmID: RNA Virus RdRP Report

`palmID` is a contained analysis suite for viral RNA-dependent RNA polymerases (RdRP) based on the “Palmprint” RNA virus barcodes described by Babaian and Edgar, 2021.

## Initialize


```
# INITIALIZE PALMID WORKSPACE -------------------
# library("palmid")

# Establish Serratus server connection
con <- SerratusConnect()

# Input file
# Generated within `palmid` container
# params are defined in YAML header to expose to CLI
input.path      <- params$input.path

if (is.null(input.path)) {
  stop("Error: No input provided.")
}

  input.fa      <- paste0(input.path, '.input.fa')   # palmscan-palmprint sequence
  input.pp      <- paste0(input.path, '.trim.fa')    # palmscan-palmprint sequence
  input.fev     <- paste0(input.path, '.fev')    # palmscan .fev report
  input.rep     <- paste0(input.path, '.txt')    # palmscan text motif-report
  input.pro     <- paste0(input.path, '.pro')    # diamond palmDB-alignment file
  input.msa     <- paste0(input.path, '.msa.fa') # muscle msa

# Output HTML Report
output.html   <- paste0(input.path, '.html')
save.plots    <- FALSE # save individual plots as png

# Parameters
  id_threshold    <- 0   # Minimum AA% to retain a hit
  max_palmdb_hits <- 500 # Maximum number of alignment hits in PalmDB hits to return

run.time <- Sys.time()

# IMPORT DATASETS -------------------------------
# Import a palmprint-analysis
pp.in <- read.fev(input.fev, FIRST = TRUE)

# Import a diamond-aligned pro file
pro.df <- read.pro(input.pro)
  # Populate with Nickname/Taxonomy-data
  pro.df$nickname     <- get.nickname(pro.df$sseqid, con, ordinal = T)
  pro.df     <- get.proTax(pro.df, con)
  
# Set backstop when too many similar hits come up
palmdb.hits <- length(pro.df$qseqid)
if (palmdb.hits > max_palmdb_hits) {
  pro.df <- pro.df[ 1:max_palmdb_hits, ]
  print.hitn <- paste0("Reporting Top ", max_palmdb_hits, "/", palmdb.hits, " matches")
} else {
  print.hitn <-  paste0("Reporting all ", palmdb.hits, " matches")
}

# SQL-Import of palmprint/sra meta-data
  # parent/child sOTU lookup, sra, biosample, date, organism, geo
palm.sra <- get.palmSra(pro.df, con)

# SQL-import of STAT kmer taxonomic analysis of the retrieved
# matching SRA libraries
stat.sra <- get.sraSTAT(palm.sra$run_id, con)
# Populate stat.sra with percent identity from palm.sra
  stat.sra$pident <- palm.sra$pident[ match(stat.sra$run_id, palm.sra$run_id) ]
  

# GENERATE REPORT-PLOTS -------------------------
# Palmprint Report
pp.report <- PlotReport(pp.in, palmdb)

# Diamond-palmDB Alignment Report
pro.report <- PlotProReport(pro.df, html = T)

# PalmDB Viral Taxonomy Report
tax.report <- PlotTaxReport(pro.df)

# Geospatial distribution Report
geo.report  <- PlotGeo2(palm.sra)
date.report <- PlotTimeline(palm.sra)

# Host/Library organism Report
orgn.report <- PlotOrgn(palm.sra, freq = FALSE)
stat.report <- PlotSTAT(stat.sra)

# Print out version
cat( paste0("palmID Version: ", params$palmid.version) )
```


```
palmID Version: 0.0.4
```


#### Summary


```
## Palmprint Detection 

  RNA-dependent RNA Polymerase detected within input sequence.

  The 99-aa palmprint barcode has a score of 51.4 (high-confidence).

## palmDB Search

  There are 66 palmprints matching the input sequence in palmDB.
  Reporting all matches with an average aa-identity of 37.9% (28.7 - 99%).

  Top match is 'u6346 (skinnyVaisya)' at 99% aa-identity 
  with an alignment CIGAR 99M, and a taxonomy of
    species: unclassified
    family : unclassified
    phylum : unclassified

  The closest named species is u232099 - Porcine bastrovirus at 83.8% identity.

## SRA Search

  Matching palmprints identified in 306 SRA sequencing libraries.
  Top-palmprint 'u6346 (skinnyVaisya)' was found in 17 libraries,
  with top-annotation: viral metagenome
```

#### Download Data


 Download palmdb\_alignment.csv

 Download sra\_metadata.csv

## Input RdRP palmprint QC

#### palmscan motif analysis

Identification of the the core catalytic motifs A,B,C within the input sequence and reporting the “palmprint” RNA-virus barcode.


```
Input FASTA sequence:
```


```
>NP_062883.2 non-structural polyprotein [Rubella virus]
MEKLLDEVLAPGGPYNLTVGSWVRDHVRSIVEGAWEVRDVVTAAQKRAIVAVIPRPVFTQMQVSDHPALHAISRYTRRHWIEWGPKEALHVLIDPSPGLLREVARVERRWVALCLHRTARKLATALAETASEAWHADYVCALRGAPSGPFYVHPEDVPHGGRAVADRCLLYYTPMQMCELMRTIDATLLVAVDLWPVALAAHVGDDWDDLGIAWHLDHDGGCPADCRGAGAGPTPGYTRPCTTRIYQVLPDTAHPGRLYRCGPRLWTRDCAVAELSWEVAQHCGHQARVRAVRCTLPIRHVRSLQPSARVRLPDLVHLAEVGRWRWFSLPRPVFQRMLSYCKTLSPDAYYSERVFKFKNALCHSITLAGNVLQEGWKGTCAEEDALCAYVAFRAWQSNARLAGIMKGAKRCAADSLSVAGWLDTIWDAIKRFLGSVPLAERMEEWEQDAAVAAFDRGPLEDGGRHLDTVQPPKSPPRPEIAATWIVHAASEDRHCACAPRCDVPRERPSAPAGQPDDEALIPPWLFAERRALRCREWDFEALRARADTAAAPAPPAPRPARYPTVLYRHPAHHGPWLTLDEPGEADAALVLCDPLGQPLRGPERHFAAGAHMCAQARGLQAFVRVVPPPERPWADGGARAWAKFFRGCAWAQRLLGEPAVMHLPYTDGDVPQLIALALRTLAQQGAALALSVRDLPGGAAFDANAVTAAVRAGPRQSAAASPPPGDPPPPRRARRSQRHSDARGTPPPAPARDPPPPAPSPPAPPRAGDPVPPIPAGPADRARDAELEVACEPSGPPTSTRADPDSDIVESYARAAGPVHLRVRDIMDPPPGCKVVVNAANEGLLAGSGVCGAIFANATAALAANCRRLAPCPTGEAVATPGHGCGYTHIIHAVAPRRPRDPAALEEGEALLERAYRSIVALAAARRWACVACPLLGAGVYGWSAAESLRAALAATRTEPVERVSLHICHPDRATLTHASVLVGAGLAARRVSPPPTEPLASCPAGDPGRPAQRSASPPATPLGDATAPEPRGCQGCELCRYTRVTNDRAYVNLWLERDRGATSWAMRIPEVVVYGPEHLATHFPLNHYSVLKPAEVRPPRGMCGSDMWRCRGWHGMPQVRCTPSNAHAALCRTGVPPRASTRGGELDPNTCWLRAAANVAQAARACGAYTSAGCPKCAYGRALSEARTHEDFAALSQRWSASHADASPDGTGDPLDPLMETVGCACSRVWVGSEHEAPPDHLLVSLHRAPNGPWGVVLEVRARPEGGNPTGHFVCAVGGGPRRVSDRPHLWLAVPLSRGGGTCAATDEGLAQAYYDDLEVRRLGDDAMARAALASVQRPRKGPYNIRVWNMAAGAGKTTRILAAFTREDLYVCPTNALLHEIQAKLRARDIDIKNAATYERRLTKPLAAYRRIYIDEAFTLGGEYCAFVASQTTAEVICVGDRDQCGPHYANNCRTPVPDRWPTERSRHTWRFPDCWAARLRAGLDYDIEGERTGTFACNLWDGRQVDLHLAFSRETVRRLHEAGIRAYTVREAQGMSVGTACIHVGRDGTDVALALTRDLAIVSLTRASDALYLHELEDGSLRAAGLSAFLDAGALAELKEVPAGIDRVVAVEQAPPPLPPADGIPEAQDVPPFCPRTLEELVFGRAGHPHYADLNRVTEGEREVRYMRISRHLLNKNHTEMPGTERVLSAVCAVRRYRAGEDGSTLRTAVARQHPRPFRQIPPPRVTAGVAQEWRMTYLRERIDLTDVYTQMGVAARELTDRYARRYPEIFAGMCTAQSLSVPAFLKATLKCVDAALGPRDTEDCHAAQGKAGLEIRAWAKEWVQVMSPHFRAIQKIIMRALRPQFLVAAGHTEPEVDAWWQAHYTTNAIEVDFTEFDMNQTLATRDVELEISAALLGLPCAEDYRALRAGSYCTLRELGSTETGCERTSGEPATLLHNTTVAMCMAMRMVPKGVRWAGIFQGDDMVIFLPEGARSAALKWTPAEVGLFGFHIPVKHVSTPTPSFCGHVGTAAGLFHDVMHQAIKVLCRRFDPDVLEEQQVALLDRLRGVYAALPDTVAANAAYYDYSAERVLAIVRELTAYARGRGLDHPATIGALEEIQTPYARANLHDAD
```


```
palmprint sequence:
```


```
>NP_062883.2
IEVDFTEFDMNQTLATRDVELEISAALLGLPCAEDYRALRAGSYCTLRELGSTETGCERTSGEPATLLHNTTVAMCMAMR
MVPKGVRWAGIFQGDDMVI
```


```
catalytic-motifs:
```


```
>NP_062883.2
   A:258-269(13.9)      B:318-331(14.0)      C:349-356(13.5)
   IEVDFTEFDMNQ    <48> SGEPATLLHNTTVA  <17> FQGDDMVI  [99]
   +|.||| ||  |         |||| | | || +        | ||||||
   lenDyskFDksq         SGdanTslGNTltn       vsGDDsvv
Score 51.4, high-confidence-RdRP: high-PSSM-score.reward-DDGGDD.good-segment-length.
```

#### palmprint QC-report

Quality control metrics on the motif-scores and size distribution of the “palmprint” informs the confidence that the input sequence contains a viral RdRP. Input scores are shown compared to score distribution from 15,010 canonical viral RdRP from GenBank.


```
# Palmscan QC-plot
plot(pp.report)
```

## Input Palmprint vs. PalmDB Analysis

#### Multiple Sequence Alignment

A `MUSCLE`-generated multiple sequence alignment of the top-10 matching hits in `palmdb`.


```
# Multiple Sequence Alignment of Top-10 hits in palmDB
cat(paste0(readLines(input.msa), collapse = "\n"))
```


```
>u80864_37.0
GYMDGTQFDSCQNAFTQEIERNILSR-LGMPTEAIEAYYSIRNNYLLSSSTFSAL-IDAAKTSGEPGTLLLNTILMMCLTAWLLRTKTVHSVIIGQGDDCFI
>u131679_75.8
VEVDFTEFDRNQTLATRDLEVEVTSHLLGFDC--LDDYKALRAGSYCQLRDLAQTETGCERTSGEPATLLHNTIVGMAM-AMRMVPPGLPWAGIFQGDDMVL
>u196907_72.7
VEVDFTEFDRNQTLATRDLEVEVTSSILGFDN--ADDYKALRAGSYCQLRDIASTETGCERTSGEPATLLHNTVVGMAM-AMRMVMSGAAWSGIFQGDDMVL
>u17187_76.8
VEVDFTEFDMHQSLATRDVELEISASLLGLPS--AEDYRALRSGSYCLIRDVAHTETGCERTSGEPATLLHNTLVATVM-AMRMCSRCRGWAGVFQGDDMVL
>NP_062883.2
IEVDFTEFDMNQTLATRDVELEISAALLGLPC--AEDYRALRAGSYCTLRELGSTETGCERTSGEPATLLHNTTVAMCM-AMRMVPKGVRWAGIFQGDDMVI
>u6346_99.0
IEVDFTEFDMNQTLATRDVELEISAALLGLPC--AEDYRALRAGSYCTLRDLGSTETGCERTSGEPATLLHNTTVAMCM-AMRMVPKGVRWAGIFQGDDMVI
>u24856_86.9
VEVDFTEFDMNQTLATRDVEAEISASLLGLPS--IEDYKALRAGSYCLLRDIASTETGCERTSGEPATLLHNTLVGMCM-AMRMVPKGVRWAGIFQGDDMVM
>u232099_83.8
VEVDFSEFDMNQTLATRDVEIEVSASLLGLPS--ADDYRALRAGSYCLLRDVAATETGCERTSGEPATLLHNTLVGMCM-AMRMVPKGVRWHGIFQGDDMVL
>u206482_37.4
LANDFSEFDSTQGVVTTKLEQKLWRH-CGLPAHLVSLYGVLRASWRIKVPGVASVQNGDMRHSGEPFTLLGNTAVNMAVSGVIMQCQGFSFAG-FKGDDSIV
>u239943_38.8
VCNDFTEFDSTQTDTTVAFEANLLRW-LGVPDCVAQLYVALRAAARVECSMLGVNAPNA-RMSGEANTLFGNTVVTMAV-NALLFPGKFGWAA-FKGDDSIV
>u168864_37.8
CCNDFTEFDSTQNSATATFECRLLRK-LGVPDGVVELYREMRAQFRVLGRDYTYT-TQWNRASGEPNTLFGNTLVTMAVNGVMSLGKDYHCA-MFKGDDSLV
```

#### Input palmprint alignment to palmDB

Input “palmprint” are aligned against `palmdb` using `diamond` to retrieve related viruses. Upto the top 500 related viruses are reported.


```
# Protein-alignment of Input vs. palmDB
# Taxonomic Demarcations (~)
# Species >90%   - Red
# Genus   70-90% - Orange
# Family  45-70% - Green
# Phylum  <45%   - Purple

plotly::ggplotly(pro.report)
```

#### Virus Taxonomy of PalmDB matches

Species-, Genus- and Phylum- level taxonomy of the matching hits in PalmDB allow for taxonomic-inference of the input virus.


```
# Viral Taxonomy of palmDB Hits
plot(tax.report)
```

#### palmDB Alignment table


Download palmDB Alignment Table

## SRA Meta-data Analysis

RNA viruses matching input are cross-referenced against 5.7M SRA sequencing libraries to identify sra-virus matches and their associated meta-data.

#### Geospatial distribution of related RNA viruses

Distribution of matching RNA viruses. Click to explore each matching record. Species, genus, family and phylum level matches are red, orange, green and purple, respectively.


```
# Geo-distribution of palmprint-containing SRA
# Taxonomic Demarcations (~)
# Species >90%   - Red
# Genus   70-90% - Orange
# Family  45-70% - Green
# Phylum  <45%   - Purple

geo.report
```

#### Virus-Organism Associations

Organism meta-data from the SRA sequencing libraries matching the input-virus. Word size and color is scaled by proximity of the input-virus to its match in the library. Wordclouds show a) Researcher provided annotations of the libraries or b) STAT automated taxonomic (orders) analysis of the reads contained within the library (Katz et al., 2021).


```
# Plot SRA  Wordcloud - id
# (scaled by AA% proximity to input palmprint)
plot(orgn.report)
```


```
# Plot SRA-Organism Wordcloud - freq
# (scaled by frequency in the SRA)
plot(stat.report)
```

#### Input Alignment to SRA-RdRp Table


Download SRA Metadata Table

#### Footer

- Source code
- Submit issue / ask question

To save this report: `File` –> `Save Page As`.

LS0tCnRpdGxlOiAicGFsbUlEOiBSTkEgVmlydXMgUmRSUCBSZXBvcnQiCm91dHB1dDoKICBodG1sX25vdGVib29rOgogICAgY29kZV9mb2xkaW5nOiBoaWRlCnBhcmFtczoKICBwYWxtaWQudmVyc2lvbjogJzAuMC40JwogIGlucHV0LnBhdGg6ICdpbnN0L2V4dGRhdGEvd2F4c3lzJwogIHByb2QucnVuOiBGQUxTRQotLS0KCmBwYWxtSURgIGlzIGEgY29udGFpbmVkIGFuYWx5c2lzIHN1aXRlIGZvciB2aXJhbCBSTkEtZGVwZW5kZW50IFJOQSBwb2x5bWVyYXNlcwooUmRSUCkgYmFzZWQgb24gdGhlICJQYWxtcHJpbnQiIFJOQSB2aXJ1cyBiYXJjb2RlcyBkZXNjcmliZWQgYnkgW0JhYmFpYW4gYW5kIEVkZ2FyLCAyMDIxXShodHRwczovL3d3dy5iaW9yeGl2Lm9yZy9jb250ZW50LzEwLjExMDEvMjAyMS4wMy4wMi40MzM2NDh2MSkuCgojIyBJbml0aWFsaXplCgpgYGB7ciBzZXR1cCwgaW5jbHVkZT1GQUxTRX0KIyBSTWFya2Rvd24gU2V0dGluZyBJbml0aWFsaXphdGlvbgojIENvbW1hbmQgTGluZSBJbnRlcmZhY2UgLS0tLQoKIyBSc2NyaXB0IC1lICJybWFya2Rvd246OnJlbmRlciggXAojICAgaW5wdXQgPSAncGFsbWlkX2Rldi5SbWQnLCBcCiMgICBvdXRwdXRfZmlsZSA9ICdhbWV4bnYuaHRtbCcsIFwKIyAgIG91dHB1dF9mb3JtYXQgPSAnaHRtbF9ub3RlYm9vaycsIFwKIyAgIHBhcmFtcz1saXN0KCBpbnB1dC5wYXRoID0gJ2FtZXhudi9hbWV4bnYnKSkiCgojIC0tLS0tLS0tLS0tLS0tLS0tLS0tLS0tLS0tLQoKa25pdHI6Om9wdHNfY2h1bmskc2V0KHdhcm5pbmcgPSBGQUxTRSwgbWVzc2FnZSA9IEZBTFNFKQpgYGAKCmBgYHtjc3MsIGVjaG89RkFMU0V9Ci8qIE1vdmUgY29kZSBmb2xkaW5nIGJ1dHRvbnMgdG8gdGhlIGxlZnQgKi8KZGl2LmNvbC1tZC0xMiAucHVsbC1yaWdodCB7CiAgZmxvYXQ6IGxlZnQgIWltcG9ydGFudAp9CmBgYAoKYGBge3IsIGluY2x1ZGU9RkFMU0UsIHdhcm5pbmc9RkFMU0V9CiMgUnVuIGFzIFByb2R1Y3Rpb24gUGlwZWxpbmUgb3IgRGV2ZWxvcG1lbnQKcHJvZHVjdGlvbi52ZXJzaW9uID0gcGFyYW1zJHByb2QucnVuCgppZiAocHJvZHVjdGlvbi52ZXJzaW9uKSB7CiAgIyBMb2FkIHN0YWJsZSBwYWxtaWQgcGFja2FnZQogIGxpYnJhcnkoJ3BhbG1pZCcsIHF1aWV0bHkgPSBUKQogIGRhdGEoInBhbG1kYiIpIAoKICAjIERpc2FibGUgd2FybmluZ3MKICAgZGVmYXVsdFcgPC0gZ2V0T3B0aW9uKCJ3YXJuIikKICAgb3B0aW9ucyh3YXJuID0gLTEpCn0gZWxzZSB7CiAgIyBXYXJuaW5ncyB2YXJpYWJsZQogIGRlZmF1bHRXIDwtIGdldE9wdGlvbigid2FybiIpCiAgb3B0aW9ucyh3YXJuID0gLTEpCgogICMgQ29tcGlsZSBhIG5ldyBwYWxtaWQgZnJvbSBzb3VyY2UKICByb3h5Z2VuMjo6cm94eWdlbmlzZSgpCiAgbG9hZCgiZGF0YS9wYWxtZGIuUkRhdGEiKQp9CgpgYGAKCgpgYGB7cn0KIyBJTklUSUFMSVpFIFBBTE1JRCBXT1JLU1BBQ0UgLS0tLS0tLS0tLS0tLS0tLS0tLQojIGxpYnJhcnkoInBhbG1pZCIpCgojIEVzdGFibGlzaCBTZXJyYXR1cyBzZXJ2ZXIgY29ubmVjdGlvbgpjb24gPC0gU2VycmF0dXNDb25uZWN0KCkKCiMgSW5wdXQgZmlsZQojIEdlbmVyYXRlZCB3aXRoaW4gYHBhbG1pZGAgY29udGFpbmVyCiMgcGFyYW1zIGFyZSBkZWZpbmVkIGluIFlBTUwgaGVhZGVyIHRvIGV4cG9zZSB0byBDTEkKaW5wdXQucGF0aCAgICAgIDwtIHBhcmFtcyRpbnB1dC5wYXRoCgppZiAoaXMubnVsbChpbnB1dC5wYXRoKSkgewogIHN0b3AoIkVycm9yOiBObyBpbnB1dCBwcm92aWRlZC4iKQp9CgogIGlucHV0LmZhICAgICAgPC0gcGFzdGUwKGlucHV0LnBhdGgsICcuaW5wdXQuZmEnKSAgICMgcGFsbXNjYW4tcGFsbXByaW50IHNlcXVlbmNlCiAgaW5wdXQucHAgICAgICA8LSBwYXN0ZTAoaW5wdXQucGF0aCwgJy50cmltLmZhJykgICAgIyBwYWxtc2Nhbi1wYWxtcHJpbnQgc2VxdWVuY2UKICBpbnB1dC5mZXYgICAgIDwtIHBhc3RlMChpbnB1dC5wYXRoLCAnLmZldicpICAgICMgcGFsbXNjYW4gLmZldiByZXBvcnQKICBpbnB1dC5yZXAgICAgIDwtIHBhc3RlMChpbnB1dC5wYXRoLCAnLnR4dCcpICAgICMgcGFsbXNjYW4gdGV4dCBtb3RpZi1yZXBvcnQKICBpbnB1dC5wcm8gICAgIDwtIHBhc3RlMChpbnB1dC5wYXRoLCAnLnBybycpICAgICMgZGlhbW9uZCBwYWxtREItYWxpZ25tZW50IGZpbGUKICBpbnB1dC5tc2EgICAgIDwtIHBhc3RlMChpbnB1dC5wYXRoLCAnLm1zYS5mYScpICMgbXVzY2xlIG1zYQoKIyBPdXRwdXQgSFRNTCBSZXBvcnQKb3V0cHV0Lmh0bWwgICA8LSBwYXN0ZTAoaW5wdXQucGF0aCwgJy5odG1sJykKc2F2ZS5wbG90cyAgICA8LSBGQUxTRSAjIHNhdmUgaW5kaXZpZHVhbCBwbG90cyBhcyBwbmcKCiMgUGFyYW1ldGVycwogIGlkX3RocmVzaG9sZCAgICA8LSAwICAgIyBNaW5pbXVtIEFBJSB0byByZXRhaW4gYSBoaXQKICBtYXhfcGFsbWRiX2hpdHMgPC0gNTAwICMgTWF4aW11bSBudW1iZXIgb2YgYWxpZ25tZW50IGhpdHMgaW4gUGFsbURCIGhpdHMgdG8gcmV0dXJuCgpydW4udGltZSA8LSBTeXMudGltZSgpCgojIElNUE9SVCBEQVRBU0VUUyAtLS0tLS0tLS0tLS0tLS0tLS0tLS0tLS0tLS0tLS0tCiMgSW1wb3J0IGEgcGFsbXByaW50LWFuYWx5c2lzCnBwLmluIDwtIHJlYWQuZmV2KGlucHV0LmZldiwgRklSU1QgPSBUUlVFKQoKIyBJbXBvcnQgYSBkaWFtb25kLWFsaWduZWQgcHJvIGZpbGUKcHJvLmRmIDwtIHJlYWQucHJvKGlucHV0LnBybykKICAjIFBvcHVsYXRlIHdpdGggTmlja25hbWUvVGF4b25vbXktZGF0YQogIHByby5kZiRuaWNrbmFtZSAgICAgPC0gZ2V0Lm5pY2tuYW1lKHByby5kZiRzc2VxaWQsIGNvbiwgb3JkaW5hbCA9IFQpCiAgcHJvLmRmICAgICA8LSBnZXQucHJvVGF4KHByby5kZiwgY29uKQogIAojIFNldCBiYWNrc3RvcCB3aGVuIHRvbyBtYW55IHNpbWlsYXIgaGl0cyBjb21lIHVwCnBhbG1kYi5oaXRzIDwtIGxlbmd0aChwcm8uZGYkcXNlcWlkKQppZiAocGFsbWRiLmhpdHMgPiBtYXhfcGFsbWRiX2hpdHMpIHsKICBwcm8uZGYgPC0gcHJvLmRmWyAxOm1heF9wYWxtZGJfaGl0cywgXQogIHByaW50LmhpdG4gPC0gcGFzdGUwKCJSZXBvcnRpbmcgVG9wICIsIG1heF9wYWxtZGJfaGl0cywgIi8iLCBwYWxtZGIuaGl0cywgIiBtYXRjaGVzIikKfSBlbHNlIHsKICBwcmludC5oaXRuIDwtICBwYXN0ZTAoIlJlcG9ydGluZyBhbGwgIiwgcGFsbWRiLmhpdHMsICIgbWF0Y2hlcyIpCn0KCiMgU1FMLUltcG9ydCBvZiBwYWxtcHJpbnQvc3JhIG1ldGEtZGF0YQogICMgcGFyZW50L2NoaWxkIHNPVFUgbG9va3VwLCBzcmEsIGJpb3NhbXBsZSwgZGF0ZSwgb3JnYW5pc20sIGdlbwpwYWxtLnNyYSA8LSBnZXQucGFsbVNyYShwcm8uZGYsIGNvbikKCiMgU1FMLWltcG9ydCBvZiBTVEFUIGttZXIgdGF4b25vbWljIGFuYWx5c2lzIG9mIHRoZSByZXRyaWV2ZWQKIyBtYXRjaGluZyBTUkEgbGlicmFyaWVzCnN0YXQuc3JhIDwtIGdldC5zcmFTVEFUKHBhbG0uc3JhJHJ1bl9pZCwgY29uKQojIFBvcHVsYXRlIHN0YXQuc3JhIHdpdGggcGVyY2VudCBpZGVudGl0eSBmcm9tIHBhbG0uc3JhCiAgc3RhdC5zcmEkcGlkZW50IDwtIHBhbG0uc3JhJHBpZGVudFsgbWF0Y2goc3RhdC5zcmEkcnVuX2lkLCBwYWxtLnNyYSRydW5faWQpIF0KICAKCiMgR0VORVJBVEUgUkVQT1JULVBMT1RTIC0tLS0tLS0tLS0tLS0tLS0tLS0tLS0tLS0KIyBQYWxtcHJpbnQgUmVwb3J0CnBwLnJlcG9ydCA8LSBQbG90UmVwb3J0KHBwLmluLCBwYWxtZGIpCgojIERpYW1vbmQtcGFsbURCIEFsaWdubWVudCBSZXBvcnQKcHJvLnJlcG9ydCA8LSBQbG90UHJvUmVwb3J0KHByby5kZiwgaHRtbCA9IFQpCgojIFBhbG1EQiBWaXJhbCBUYXhvbm9teSBSZXBvcnQKdGF4LnJlcG9ydCA8LSBQbG90VGF4UmVwb3J0KHByby5kZikKCiMgR2Vvc3BhdGlhbCBkaXN0cmlidXRpb24gUmVwb3J0Cmdlby5yZXBvcnQgIDwtIFBsb3RHZW8yKHBhbG0uc3JhKQpkYXRlLnJlcG9ydCA8LSBQbG90VGltZWxpbmUocGFsbS5zcmEpCgojIEhvc3QvTGlicmFyeSBvcmdhbmlzbSBSZXBvcnQKb3Jnbi5yZXBvcnQgPC0gUGxvdE9yZ24ocGFsbS5zcmEsIGZyZXEgPSBGQUxTRSkKc3RhdC5yZXBvcnQgPC0gUGxvdFNUQVQoc3RhdC5zcmEpCgojIFByaW50IG91dCB2ZXJzaW9uCmNhdCggcGFzdGUwKCJwYWxtSUQgVmVyc2lvbjogIiwgcGFyYW1zJHBhbG1pZC52ZXJzaW9uKSApCgpgYGAKCiMjIyMgU3VtbWFyeQoKYGBge3IsIGVjaG8gPSBGQUxTRX0KIyBHZW5lcmF0ZSB0ZXh0LWJhc2VkIHN1bW1hcnkgb2YgcGFsbUlEIGRhdGEKcGFsbS5zdW1tYXJ5IDwtIHRleHRfc3VtbWFyeShwcC5pbiwgcHJvLmRmLCBwYWxtLnNyYSwgcGFsbWRiLmhpdHMpCmNhdChwYWxtLnN1bW1hcnkpCmBgYAoKIyMjIyBEb3dubG9hZCBEYXRhCgpgYGB7ciwgZWNobyA9IEZBTFNFfQpvcHRpb25zKHdhcm4gPSAtMSkKCnByby5kZiAlPiUKICBkb3dubG9hZHRoaXM6OmRvd25sb2FkX3RoaXMoCiAgICBvdXRwdXRfbmFtZSA9IHBhc3RlMChpbnB1dC5wYXRoLCAnLnBybycpLAogICAgb3V0cHV0X2V4dGVuc2lvbiA9ICIuY3N2IiwKICAgIGJ1dHRvbl9sYWJlbCA9ICJEb3dubG9hZCBwYWxtZGJfYWxpZ25tZW50LmNzdiIsCiAgICBidXR0b25fdHlwZSA9ICJzdWNjZXNzIiwKICAgIGhhc19pY29uID0gVFJVRSwKICAgIGljb24gPSAiZmEgZmEtZmlsZS1leHBvcnQiCiAgKQoKcGFsbS5zcmEgJT4lCiAgZG93bmxvYWR0aGlzOjpkb3dubG9hZF90aGlzKAogICAgb3V0cHV0X25hbWUgPSBwYXN0ZTAoaW5wdXQucGF0aCwgJy5zcmEubWV0YWRhdGEnKSwKICAgIG91dHB1dF9leHRlbnNpb24gPSAiLmNzdiIsCiAgICBidXR0b25fbGFiZWwgPSAiRG93bmxvYWQgc3JhX21ldGFkYXRhLmNzdiIsCiAgICBidXR0b25fdHlwZSA9ICJzdWNjZXNzIiwKICAgIGhhc19pY29uID0gVFJVRSwKICAgIGljb24gPSAiZmEgZmEtZmlsZS1leHBvcnQiCiAgKQpgYGAKIyMgSW5wdXQgUmRSUCBwYWxtcHJpbnQgUUMKCiMjIyMgcGFsbXNjYW4gbW90aWYgYW5hbHlzaXMKCklkZW50aWZpY2F0aW9uIG9mIHRoZSB0aGUgY29yZSBjYXRhbHl0aWMgbW90aWZzIEEsQixDIAp3aXRoaW4gdGhlIGlucHV0IHNlcXVlbmNlIGFuZCByZXBvcnRpbmcgdGhlICJwYWxtcHJpbnQiClJOQS12aXJ1cyBiYXJjb2RlLgoKYGBge3IsIGVjaG8gPSBGQUxTRX0KIyBJbnB1dCBTZXF1ZW5jZQpjYXQoIklucHV0IEZBU1RBIHNlcXVlbmNlOiBcblxuIikKY2F0KHBhc3RlMChyZWFkTGluZXMoaW5wdXQuZmEpLCBjb2xsYXBzZSA9ICJcbiIpLCAiXG4iKQpjYXQoIlxuIikKCiMgUGFsbXNjYW4tZ2VuZXJhdGVkIHJlcG9ydHMKY2F0KCJwYWxtcHJpbnQgc2VxdWVuY2U6IFxuXG4iKQpjYXQocGFzdGUwKHJlYWRMaW5lcyhpbnB1dC5wcCksIGNvbGxhcHNlID0gIlxuIiksICJcbiIpCmNhdCgiXG4iKQoKY2F0KCJjYXRhbHl0aWMtbW90aWZzOiBcbiIpCmNhdChwYXN0ZTAocmVhZExpbmVzKGlucHV0LnJlcCksIGNvbGxhcHNlID0gIlxuIiksICJcbiIpCmNhdCgiXG4iKQoKIyBsYXBwbHkoc2NhbihpbnB1dC5yZXAsICdjaGFyYWN0ZXInKSxjYXQpW1sxXV0KCmBgYAoKIyMjIyBwYWxtcHJpbnQgUUMtcmVwb3J0CgpRdWFsaXR5IGNvbnRyb2wgbWV0cmljcyBvbiB0aGUgbW90aWYtc2NvcmVzIGFuZCBzaXplIGRpc3RyaWJ1dGlvbgpvZiB0aGUgInBhbG1wcmludCIgaW5mb3JtcyB0aGUgY29uZmlkZW5jZSB0aGF0IHRoZSBpbnB1dCBzZXF1ZW5jZQpjb250YWlucyBhIHZpcmFsIFJkUlAuIElucHV0IHNjb3JlcyBhcmUgc2hvd24gY29tcGFyZWQgdG8gc2NvcmUgCmRpc3RyaWJ1dGlvbiBmcm9tIDE1LDAxMCBjYW5vbmljYWwgdmlyYWwgUmRSUCBmcm9tIEdlbkJhbmsuCgpgYGB7ciwgZmlnLmhlaWdodCA9IDQuNSwgZmlnLndpZHRoID0gOX0KIyBQYWxtc2NhbiBRQy1wbG90CnBsb3QocHAucmVwb3J0KQpgYGAKCiMjIElucHV0IFBhbG1wcmludCB2cy4gUGFsbURCIEFuYWx5c2lzCgojIyMjIE11bHRpcGxlIFNlcXVlbmNlIEFsaWdubWVudAoKQSBgTVVTQ0xFYC1nZW5lcmF0ZWQgbXVsdGlwbGUgc2VxdWVuY2UgYWxpZ25tZW50IG9mIHRoZSAKdG9wLTEwIG1hdGNoaW5nIGhpdHMgaW4gW2BwYWxtZGJgXShodHRwczovL2dpdGh1Yi5jb20vcmNlZGdhci9wYWxtZGIpLgoKYGBge3J9CiMgTXVsdGlwbGUgU2VxdWVuY2UgQWxpZ25tZW50IG9mIFRvcC0xMCBoaXRzIGluIHBhbG1EQgpjYXQocGFzdGUwKHJlYWRMaW5lcyhpbnB1dC5tc2EpLCBjb2xsYXBzZSA9ICJcbiIpKQpgYGAKCiMjIyMgSW5wdXQgcGFsbXByaW50IGFsaWdubWVudCB0byBwYWxtREIKCklucHV0ICJwYWxtcHJpbnQiIGFyZSBhbGlnbmVkIGFnYWluc3QgW2BwYWxtZGJgXShodHRwczovL2dpdGh1Yi5jb20vcmNlZGdhci9wYWxtZGIpCnVzaW5nIGBkaWFtb25kYCB0byByZXRyaWV2ZSByZWxhdGVkIHZpcnVzZXMuIFVwdG8gdGhlIHRvcCA1MDAgcmVsYXRlZCB2aXJ1c2VzCmFyZSByZXBvcnRlZC4KCgpgYGB7ciwgZmlnLmhlaWdodCA9IDUsIGZpZy53aWR0aCA9IDl9CiMgUHJvdGVpbi1hbGlnbm1lbnQgb2YgSW5wdXQgdnMuIHBhbG1EQgojIFRheG9ub21pYyBEZW1hcmNhdGlvbnMgKH4pCiMgU3BlY2llcyA+OTAlICAgLSBSZWQKIyBHZW51cyAgIDcwLTkwJSAtIE9yYW5nZQojIEZhbWlseSAgNDUtNzAlIC0gR3JlZW4KIyBQaHlsdW0gIDw0NSUgICAtIFB1cnBsZQoKcGxvdGx5OjpnZ3Bsb3RseShwcm8ucmVwb3J0KQoKYGBgCiFbXShpbWcvdGF4X2xlZ2VuZC5wbmcpCgojIyMjIFZpcnVzIFRheG9ub215IG9mIFBhbG1EQiBtYXRjaGVzCgpTcGVjaWVzLSwgR2VudXMtIGFuZCBQaHlsdW0tIGxldmVsIHRheG9ub215IG9mIHRoZSBtYXRjaGluZwpoaXRzIGluIFBhbG1EQiBhbGxvdyBmb3IgdGF4b25vbWljLWluZmVyZW5jZSBvZiB0aGUgaW5wdXQKdmlydXMuCgpgYGB7ciwgZmlnLmhlaWdodD0xMCwgZmlnLndpZHRoPTE2LCByZXRpbmEgPSAxfQojIFZpcmFsIFRheG9ub215IG9mIHBhbG1EQiBIaXRzCnBsb3QodGF4LnJlcG9ydCkKYGBgCiMjIyMgcGFsbURCIEFsaWdubWVudCB0YWJsZQoKYGBge3IsIGVjaG8gPSBGQUxTRX0KIyMjIERhdGEtVGFibGU6IGBwYWxtREIuY3N2YAojIERhdGEgdGFibGUgZm9yIHBhbG1EQiBBbGlnbm1lbnQKCmJsYXN0LmNvbCA8LSBsaW5rQkxBU1QocHJvLmRmJHNzZXFpZCwgcHJvLmRmJGZ1bGxfc3NlcSkKCmNiaW5kKCBwcm8uZGZbLCBjKCJzc2VxaWQiLCAibmlja25hbWUiLCAidHNwZSIsICJwaWRlbnQiLCAiZXZhbHVlIiwgImNpZ2FyIildLAogICAgICAgYmxhc3QuY29sKSAlPiUKICBEVDo6ZGF0YXRhYmxlKAogICAgcm93bmFtZXMgPSBGQUxTRSwgZmlsdGVyID0gInRvcCIsIGVzY2FwZSA9IEZBTFNFLAogICAgb3B0aW9ucyA9IGxpc3QocGFnZUxlbmd0aCA9IDEwLCBzY3JvbGxYID0gVFJVRSkKICApCgpgYGAKW0Rvd25sb2FkIHBhbG1EQiBBbGlnbm1lbnQgVGFibGVdKCNkb3dubG9hZC1kYXRhKQoKIyMgU1JBIE1ldGEtZGF0YSBBbmFseXNpcwoKUk5BIHZpcnVzZXMgbWF0Y2hpbmcgaW5wdXQgYXJlIGNyb3NzLXJlZmVyZW5jZWQgYWdhaW5zdAo1LjdNIFNSQSBzZXF1ZW5jaW5nIGxpYnJhcmllcyB0byBpZGVudGlmeSBzcmEtdmlydXMgbWF0Y2hlcwphbmQgdGhlaXIgYXNzb2NpYXRlZCBtZXRhLWRhdGEuCgojIyMjIEdlb3NwYXRpYWwgZGlzdHJpYnV0aW9uIG9mIHJlbGF0ZWQgUk5BIHZpcnVzZXMKCkRpc3RyaWJ1dGlvbiBvZiBtYXRjaGluZyBSTkEgdmlydXNlcy4gQ2xpY2sgdG8gZXhwbG9yZQplYWNoIG1hdGNoaW5nIHJlY29yZC4gU3BlY2llcywgZ2VudXMsIGZhbWlseSBhbmQgcGh5bHVtCmxldmVsIG1hdGNoZXMgYXJlIHJlZCwgb3JhbmdlLCBncmVlbiBhbmQgcHVycGxlLCByZXNwZWN0aXZlbHkuCgpgYGB7ciwgZmlnLmhlaWdodCA9IDYsIGZpZy53aWR0aCA9IDl9CiMgR2VvLWRpc3RyaWJ1dGlvbiBvZiBwYWxtcHJpbnQtY29udGFpbmluZyBTUkEKIyBUYXhvbm9taWMgRGVtYXJjYXRpb25zICh+KQojIFNwZWNpZXMgPjkwJSAgIC0gUmVkCiMgR2VudXMgICA3MC05MCUgLSBPcmFuZ2UKIyBGYW1pbHkgIDQ1LTcwJSAtIEdyZWVuCiMgUGh5bHVtICA8NDUlICAgLSBQdXJwbGUKCmdlby5yZXBvcnQKYGBgCiFbXShpbWcvdGF4X2xlZ2VuZC5wbmcpCgpgYGB7ciwgZWNobyA9IEZBTFNFLCBmaWcuaGVpZ2h0ID0gMywgZmlnLndpZHRoID0gOX0KIyBHZW8tZGlzdHJpYnV0aW9uIG9mIHBhbG1wcmludC1jb250YWluaW5nIFNSQQpwbG90bHk6OmdncGxvdGx5KGRhdGUucmVwb3J0KQpgYGAKCiMjIyMgVmlydXMtT3JnYW5pc20gQXNzb2NpYXRpb25zCgpPcmdhbmlzbSBtZXRhLWRhdGEgZnJvbSB0aGUgU1JBIHNlcXVlbmNpbmcgbGlicmFyaWVzIG1hdGNoaW5nIHRoZSBpbnB1dC12aXJ1cy4KV29yZCBzaXplIGFuZCBjb2xvciBpcyBzY2FsZWQgYnkgcHJveGltaXR5IG9mIHRoZSBpbnB1dC12aXJ1cyB0byBpdHMgbWF0Y2ggaW4gCnRoZSBsaWJyYXJ5LiBXb3JkY2xvdWRzIHNob3cgYSkgUmVzZWFyY2hlciBwcm92aWRlZCBhbm5vdGF0aW9ucyBvZiB0aGUgbGlicmFyaWVzCm9yIGIpIFNUQVQgYXV0b21hdGVkIHRheG9ub21pYyAob3JkZXJzKSBhbmFseXNpcyBvZiB0aGUgcmVhZHMgY29udGFpbmVkIHdpdGhpbgp0aGUgbGlicmFyeSAoW0thdHogZXQgYWwuLCAyMDIxXShodHRwczovL2dlbm9tZWJpb2xvZ3kuYmlvbWVkY2VudHJhbC5jb20vYXJ0aWNsZXMvMTAuMTE4Ni9zMTMwNTktMDIxLTAyNDkwLTApKS4KCmBgYHtyLCBmaWcud2lkdGg9OCwgZmlnLmhlaWdodD01LCByZXRpbmEgPSAyfQojIFBsb3QgU1JBICBXb3JkY2xvdWQgLSBpZAojIChzY2FsZWQgYnkgQUElIHByb3hpbWl0eSB0byBpbnB1dCBwYWxtcHJpbnQpCnBsb3Qob3Jnbi5yZXBvcnQpCmBgYAoKYGBge3IsIGZpZy53aWR0aD04LCBmaWcuaGVpZ2h0PTUsIHJldGluYSA9IDJ9CiMgUGxvdCBTUkEtT3JnYW5pc20gV29yZGNsb3VkIC0gZnJlcQojIChzY2FsZWQgYnkgZnJlcXVlbmN5IGluIHRoZSBTUkEpCnBsb3Qoc3RhdC5yZXBvcnQpCmBgYAohW10oaW1nL3N0YXRfbGVnZW5kLnBuZykKCiMjIyMgSW5wdXQgQWxpZ25tZW50IHRvIFNSQS1SZFJwIFRhYmxlCgpgYGB7ciwgZWNobyA9IEZBTFNFfQojIyMgRGF0YS1UYWJsZTogYHNyYS5tZXRhZGF0YS5jc3ZgCiMgRGF0YSB0YWJsZSBmb3IgcGFsbURCIEFsaWdubWVudAoKYmxhc3QuY29sIDwtIGxpbmtCTEFTVChwYWxtLnNyYSRzcmFfc2VxdWVuY2UsCiAgICAgICAgICAgICAgICAgICAgICAgcGFzdGUwKHBhbG0uc3JhJHJ1bl9pZCwiXyIscGFsbS5zcmEkcGFsbV9pZCkKICAgICAgICAgICAgICAgICAgICAgICApCgpjYmluZCggcGFsbS5zcmFbLCBjKCJydW5faWQiLCAiYmlvc2FtcGxlX2lkIiwgJ3BhbG1faWQnLCAncGlkZW50JywgJ2NvdmVyYWdlJywgJ2V2YWx1ZScsICdzY2llbnRpZmljX25hbWUnKV0sCiAgICAgICBibGFzdC5jb2wpICU+JQogIERUOjpkYXRhdGFibGUoCiAgICByb3duYW1lcyA9IEZBTFNFLCBmaWx0ZXIgPSAidG9wIiwgZXNjYXBlID0gRiwKICAgIG9wdGlvbnMgPSBsaXN0KHBhZ2VMZW5ndGggPSAxMCwgc2Nyb2xsWCA9IFQpCiAgKQoKYGBgCgpbRG93bmxvYWQgU1JBIE1ldGFkYXRhIFRhYmxlXSgjZG93bmxvYWQtZGF0YSkKCiMjIyMgRm9vdGVyCgotIFtTb3VyY2UgY29kZV0oaHR0cHM6Ly9naXRodWIuY29tL2FiYWJhaWFuL3BhbG1pZCkKLSBbU3VibWl0IGlzc3VlIC8gYXNrIHF1ZXN0aW9uXShodHRwczovL2dpdGh1Yi5jb20vYWJhYmFpYW4vcGFsbWlkL2lzc3VlcykKClRvIHNhdmUgdGhpcyByZXBvcnQ6IGBGaWxlYCAtLT4gYFNhdmUgUGFnZSBBc2AuCgpgYGB7ciwgaW5jbHVkZT1GQUxTRX0KaWYgKHNhdmUucGxvdHMpIHsKICAKICAjIFNBVkUgUFAtUkVQT1JUCiAgcG5nKGZpbGVuYW1lID0gb3V0cHV0LnJlcG9ydCwgd2lkdGggPSA4MDAsIGhlaWdodCA9IDQwMCkKICAgcGxvdChwcC5yZXBvcnQpCiAgZGV2Lm9mZigpCiAgCiAgIyBTQVZFIFBSTy1SRVBPUlQgCiAgcG5nKGZpbGVuYW1lID0gb3V0cHV0LnBybywgd2lkdGggPSA4MDAsIGhlaWdodCA9IDQwMCkKICAgIHBsb3QocHJvLnJlcG9ydCkKICBkZXYub2ZmKCkKICAKICAjIFNBVkUgVEFYLVJFUE9SVAogIGdnc2F2ZShvdXRwdXQudGF4LAogICAgcGxvdCh0YXgucmVwb3J0KSwKICAgIHdpZHRoID0gMTYsCiAgICBoZWlnaHQgPSAxMCwKICAgIGRwaSA9IDcyCiAgKQogIAogICMgU0FWRSBHRU8tUkVQT1JUCiAgaHRtbHdpZGdldHM6OnNhdmVXaWRnZXQoZ2VvLnJlcG9ydCwgZmlsZSA9IG91dHB1dC5nZW8pCgogICMgU0FWRSBPUkdOLVJFUE9SVAogIHBuZyhmaWxlbmFtZSA9IG91dHB1dC5vcmduLCB3aWR0aCA9IDgwMCwgaGVpZ2h0ID0gNDAwLCByZXMgPSAxMDApCiAgICBwbG90KG9yZ24ucmVwb3J0KQogIGRldi5vZmYoKQoKfQoKYGBgCgpgYGB7ciwgaW5jbHVkZSA9IEZBTFNFfQojIERldmVsb3BtZW50IENvZGUKI3JtYXJrZG93bjo6cmVuZGVyKCdwYWxtaWRfZGV2LlJtZCcsCiMgICAgICAgICAgICAgICAgICBvdXRwdXRfZmlsZSA9IG91dHB1dC5odG1sKQoKIyAjIEdlbmVyYXRlIC9kYXRhIGZpbGVzCiMgIyBiYXNlZCBvbiB3YXhzeXMgZXhhbXBsZQojIHdheHN5cy5wYWxtLnNyYSA8LSBwYWxtLnNyYQojIHdheHN5cy5wYWxtcHJpbnQgPC0gcHAuaW4KIyB3YXhzeXMucHJvLmRmIDwtIHByby5kZgojIHdheHN5cy5zdGF0LnNyYSA8LSBzdGF0LnNyYQojIAojIHNhdmUoIHdheHN5cy5wYWxtLnNyYSwgZmlsZSA9ICJkYXRhL3dheHN5cy5wYWxtLnNyYS5SRGF0YSIsIGNvbXByZXNzID0gInh6IikKIyBzYXZlKCB3YXhzeXMucGFsbXByaW50LCBmaWxlID0gImRhdGEvd2F4c3lzLnBhbG1wcmludC5SRGF0YSIsIGNvbXByZXNzID0gInh6IikKIyBzYXZlKCB3YXhzeXMucHJvLmRmLCBmaWxlID0gImRhdGEvd2F4c3lzLnByby5kZi5SRGF0YSIsIGNvbXByZXNzID0gInh6IikKIyBzYXZlKCB3YXhzeXMuc3RhdC5zcmEsIGZpbGUgPSAiZGF0YS93YXhzeXMuc3RhdC5zcmEuUkRhdGEiLCBjb21wcmVzcyA9ICJ4eiIpCmBgYAoK
